# Investigating the Rheological Properties of Carbon Nanotubes/Polymer Composites Modified Asphalt

**DOI:** 10.3390/ma13184077

**Published:** 2020-09-14

**Authors:** Ming Liang, Linping Su, Peizhao Li, Jingtao Shi, Zhanyong Yao, Jizhe Zhang, Hongguang Jiang, Weixin Luo

**Affiliations:** 1 School of Qilu Transportation, Shandong University, Jinan 250002, China; ming.liang@sdu.edu.cn (M.L.); 201914986@mail.sdu.edu.cn (L.S.); 201814689@mail.sdu.edu.cn (P.L.); zhanyong-y@sdu.edu.cn (Z.Y.); jizhe.zhang@sdu.edu.cn (J.Z.); 2 PetroChina Fuel Oil Co., Ltd., Research Institute, Beijing 100195, China; sjt0822@163.com; 3 State Key Laboratory of Special Functional Waterproof Materials, Beijing Oriental Yuhong Waterproof Technology Co. Ltd., Beijing 100123, China

**Keywords:** modified asphalt, carbon nanotube, polyethylene, rheological properties, micro-morphologies

## Abstract

The utilization of nanomaterials in the field of binder materials for road paving has attracted researchers’ attention in recent years. This study presented the performance properties of a binder modified with carbon nanotubes (CNT) and polyethylene (PE). The rheological properties, adhesion behavior, morphology, and storage stability of the modified asphalt were investigated. Experimental analysis indicated a positive effect of CNT/PE composites on the performance of the binder. The results indicate that the combined use of CNT and PE shows a significant enhancement on complex modulus, viscosity, and creep recovery of the binder at high temperatures and a great decrease in compliance, indicating great resistance to permanent deformation. Meanwhile, only using CNT to improve the high temperature performance of the binder is limited due to high shear mixing. CNT/PE modifiers enhance the cracking resistance at low temperatures and moisture damage resistance. The CNT/PE melt mixing composites endow asphalt with stronger cracking resistance, better resistance to moisture damage and workability. Asphalt with CNT/PE composites formed an even dispersion system. Notably, CNT bridges on the interface between PE phase and asphalt for the two modified asphalts, which reinforces the cohesion of interface. Asphalt with CNT/PE composites showed improved storage stability in comparison with PE modified asphalt.

## 1. Introduction

Increased traffic-related factors on asphalt pavement, including heavier loads, higher traffic volume combined with adverse climate variation, have resulted in the increasingly stringent performance requirement of asphalt binders. To obtain asphalt with high quality, polymer modification for asphalt is commonly used in application [1]. Notably, in consideration of the significantly increased rutting on the pavement, polyethylene (PE) modification for asphalt has become increasingly popular in recent years [2,3,4]. However, polymer modified asphalt also has some drawbacks, such as poor solubility of polymers, poor storage stability, susceptibility to separation at high temperatures, and low aging resistance, among others. Several techniques to remove these drawbacks, including vulcanization, saturation, adding antioxidants, and using clay minerals, have been developed [5]. In addition to those attempts, some researchers have begun to turn their attention towards nanotechnology [6].

Nanotechnology controls matter at the atomic and molecular level and deals with the creation and use of materials or devices with the size of 100 nm or smaller. Thus, nanotechnology has significantly changed the general views on materials’ properties. Nanomaterials have been used with wide-ranging applications in recent years to improve the properties of various materials. Among various nano-sized materials, carbon nanotubes (CNT) appear to be the most promising additive for performance reinforcement of structural and construction materials. The molecular structure of CNT presents a seamless hollow cylinder with the diameter of 1 nm that is rolled up by one-atom thick sheet of graphite [7]. CNT shows superior mechanical properties compared with other engineering materials. Young’s modulus of CNT can reach up to 1000 GPa and tensile strength can reach 150 GPa. As a result, CNT has the potential to improve the performance of various materials. 

The use of CNT in the area of binder materials in road paving applications has attracted researcher’s attention in recent years. Some researchers reported the application of CNT as reinforcing additives for asphalt binder modification. Xiao et al. [8,9] found that CNT improves the resistance to permanent deformation of aged binders, especially at high dosage. Amirkhanian et al. [10,11] showed that the rutting and fatigue resistance of asphalt can be enhanced by adding sufficiently high percentage (1%) of CNT. Ziari et al. [12,13] reported that the incorporation of CNT leads to the increased complex modulus and physical properties of asphalt binders. Regarding to asphalt mixtures, Ameri et al. [14] found that CNT can enhance both fracture resistance and fatigue performance of asphalt mixtures when using in a sufficiently high dosage. Santagata et al. [15,16] presented the enhanced fatigue and healing behaviors of asphalt containing CNT. In terms of processing technology, Santagata et al. [17,18] also reported the effect of sonication processing on rheological properties of asphalt. They believed that sonication is a key factor in order to obtain well-dispersed CNT asphalt, which is important for the enhancement of fatigue life of asphalt binders. However, these reports recommended the use of a relatively high dosage of CNT (>1%) to improve the performance of asphalt. Considering the high price of CNT, asphalt modified by a high percentage of CNT may become less attractive. From this perspective, the combination of polymer and CNT for asphalt modification appears to be a more promising approach to obtain asphalt binders with high quality. Only a few studies conducted the polymer/CNT composite modification for asphalt. Goli et al. [19] investigated the influence of CNT on physical, rheological properties and storage stability of SBS (styrene–butadiene–styrene) modified asphalt. The results showed that the addition of CNT to SBS modified asphalt improves the storage stability. Wang et al. [20] found that the resistance to aging of SBS modified asphalt was enhanced by adding a suitable amount of CNT and CNT enriched the interphase. Tsantilis et al. [21] investigated the rheological properties of ternary composites constituted by bitumen, carbon nanotubes additives and SBS polymer modifiers. The results indicated that CNT acted as compatibility activators between bitumen and linear SBS, whereas benefits of modification were jeopardized in blends prepared with CNTs and radial SBS. Our previous studies indicated that premixed CNTs/PE composites benefits for the low temperature cracking resistance and has the good anti-aging performance [22]. However, the current few studies did not deeply illustrate the interaction of polymer/CNT modified asphalt from the viewpoint of morphology, property, and field performance. Detailed information on the complicated relationships of these composites in modified binders was still scarce. Therefore, more research on the application of CNT in polymer modified asphalt needs to be carried out, in order to illustrate the effect of CNT combined polymer for asphalt modification and propose some guidelines for its use.

The primary goal of this study was to explore the effect of CNT/PE composites as reinforcement modifier additive on rheological properties, interfacial behavior, low temperature performance, bonding behavior, and storage stability of the modified asphalt. Toward this end, the commercial CNT/PE composites were used to modify base asphalt. For a comparison of CNT/PE composites, the CNT combined with PE was also added sequentially into asphalt matrix to prepare the contrast modified asphalt. High-shear mixer was employed to disperse the nanoparticle/polymer composites. Physical properties were evaluated by conventional tests. High and low temperature performances of CNT/PE modified asphalt were investigated through temperature sweep in DSR, viscosity in a Brookfield viscometer, and a direct tension tester. In addition, the interaction and interfacial properties were assessed by morphology and asphalt–mineral adhesion tests. Finally, the effect of CNT/PE composite on storage stability of the modified asphalt was evaluated by measuring the viscosity difference and separation index.

## 2. Materials and Methods

### 2.1. Materials

Neat base asphalt belonging to the standard 50/70 penetration grade was provided by a local refinery (Qilu Oil Refinery, Zibo, China). Nanoparticle/polymer (CNT/PE) composites (JCNANO Company, Nanjing, China) were supplied in a form of black pellets. The content of CNT in composites is 20 wt % and the polymer matrix (Baling Petrochemical, Yueyang, China) is commercial high-density polyethylene (HDPE). The density and melting point of commercial CNT/PE composites are 0.952 g·cm^−3^ and 145 °C. The multi-wall CNT comes from the same source with CNT/PE composites and the basic properties can be seen in Table 1. Commercial HDPE is supplied in the form of pellets. The properties of selected PE are described in Table 2.

A high-shear mixer (Weiyu-5000, Shanghai, China) was employed to prepare various CNT/PE modified asphalt. For CNT/PE melt blend composites, the composite modifier was added in hot asphalt and mixed for 30 min under the high-shearing with 4000 rpm. The blends were then mixed with a mechanical stirrer (IKA-20, Staufen, Germany) at the speed of 1500 rpm for an extra time of 60 min. The dosage of CNT/PE composite additive is 5 wt % by weight of asphalt binder and thus, the actual content of CNT in asphalt matrix is 1 wt %. Regarding to the combination of CNT and HDPE, CNT was first added and manually blended to asphalt before high-shearing conditioning. PE additive was incorporated into asphalt at the beginning of high-shearing. The contents of CNT and HDPE are 1 wt % and 4 wt % respectively. For the preparation of all samples, the temperature was set as 160 ± 1 °C and kept constant through an electric heating jacket (Hualu SKM-2, Heze, China). All samples were named by the type of modifier and dosage. The processed asphalt was a blank sample and conditioned without the presence of modifier.

### 2.2. Tests and Measurements

Basic properties of the modified asphalts were investigated by conventional physical tests, including penetration [23], softening point [24], and ductility [25]. Workability was characterized by rotational viscosity using Brookfield viscometer [26].

To determine the rheological properties, oscillatory shear tests were performed on all samples using a dynamic shear rheometer (AR2000ex, TA, New Castle, DE, USA) (DSR), obtaining complex modulus, elastic modulus and phase angle with a temperature range from 30–90 °C. High temperature performance was evaluated by multi-stress creep recovery (MSCR) tests at 0.1 and 3.2 kPa and 60 °C. For these tests on DSR, the parallel plate with a diameter of 25 mm and the thickness of modified asphalt for 1 mm were applied.

Low temperature mechanical response was investigated by direct tension test (DTT) at −12 °C. The sample preparation for DTT is similar as the specimen preparation procedure for asphalt ductility test. After cooling for 1 h in methyl acetate solution bath, the samples were stretched by applying a horizontal load with a constant displacement of 1 mm/min. Till the sample was fractured, parameters, including elongation at break, break strength, and stress–strain data, could be obtained.

The effect of CNT/PE composites on the adhesion performance was evaluated by means of aggregate-binder bonding behavior, which is identified by the bonding strength between aggregate substrate and binder using an automatic adhesion tester (Positest AT-A equipment, DeFelsko, Shanghai, China). A certain thickness of asphalt film is sandwiched between aggregate substrate and pull-stub. To ensure the good bonding of aggregate-binder, the molten sample was loaded into the space between aggregate substrate and pull-stub, then cooling down to room temperature and trimming of excessive asphalt around the edge of pull-stub. After the completion of the aggregate–binder specimen, the specimen was loaded on adhesion tester (Positest AT-A equipment, DeFelsko, Shanghai, China) and a constant rate of pulling pressure was imposed to the pull-stub. The maximum tensile strength to separate the aggregate-binder interface was detected by the Positest AT-A equipment with limestone as the aggregate substrate at room temperature.

Microstructure of the CNT/PE composite modified asphalt was investigated using scanning electron microscope (SEM) of Hitachi S4800 (Hitachi, Ltd., Tokyo, Japan). SEM samples were placed on an aluminum stub and coated with gold thin film of 0.6 nm at room temperature using the electron microscopy sciences system.

A storage stability test was carried out for CNT/PE composite modified asphalt to evaluate the influence of CNT on high temperature stability of PE binder. A certain amount of sample was poured into a tube in accordance with ASTM D5892. The test was performed by sealing the tube, placing it vertically in storage environment with the temperature of 163 °C for 48 h, then freezing it in a refrigerator for 4 h, and finally cutting into three equal pieces. In general, the difference in softening point of the top and bottom pieces of the tube is compared. It is regarded that the sample with a difference lower than 2.2 °C has good storage stability.

## 3. Results

### 3.1. Conventional Properties

**Figure 1:**
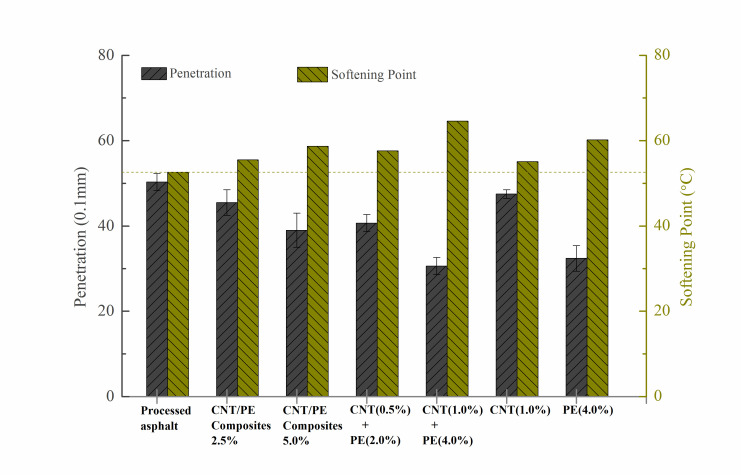

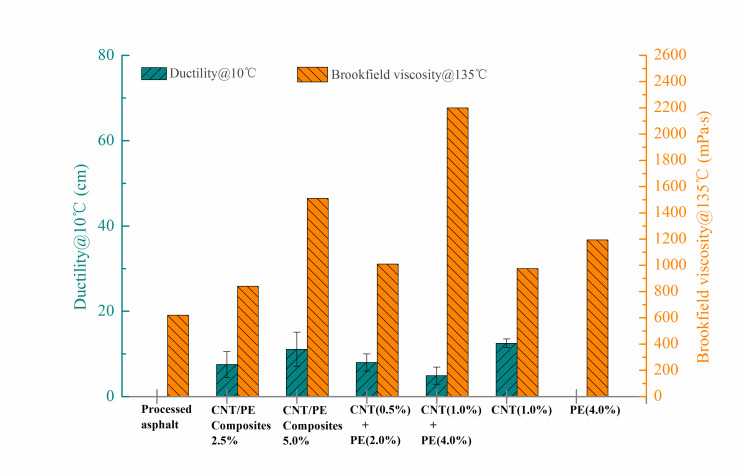
Conventional properties of modified asphalt specimen: softening point and penetration (**a**); ductility @ 10 °C and Brookfield viscosity @ 135 °C (**b**).

The effect of CNT/PE composites on the conventional properties of the modified asphalt was investigated based on softening point, penetration, ductility at 10 °C and Brookfield viscosity at 135 °C. The basic physical properties were presented in Figure 1. In comparison to processed asphalt, the incorporation of CNT/PE composites increased the softening point and decreased penetration (Figure 1a). Meanwhile, increasing the CNT/PE composites (2.5% to 5.0%) further resulted in the increase of softening point and the decrease of penetration. For the same content of modifier, the combined use of CNT and PE shows higher softening point and lower penetration, in comparison of CNT/PE composites. CNT as well as PE also improves the softening point. Compared with single CNT or single PE, the combined use of them or the composites presents an advantage in terms of high temperature performance (softening point).

As displayed in Figure 1b, the CNT/PE composite caused the increased ductility of binder. CNT appears to benefit for the ductility and PE has no obvious improvement on ductility. However, the combined use of CNT and PE shows better ductility performance comparing to single one. The CNT/PE composite leads to better ductility performance of binder with comparison of combined use of CNT and PE. Brookfield viscosity of binders improves at 135 °C whether the melt mixing CNT/PE composite or combined addition of CNT and PE. For the same dosage, the melt mixing CNT/PE composite showed decreased viscosity comparing to combined addition, indicating better workability. Therefore, the melt mixing CNT/PE composite caused the decreased softening point and the increased penetration, with comparison of simple addition of CNT and PE. Meanwhile, the melt mixing CNT/PE composite leads to better ductility performance of binder in comparison to the combined use of CNT and PE.

### 3.2. Viscosity-Temperature Property

**Figure 2:**
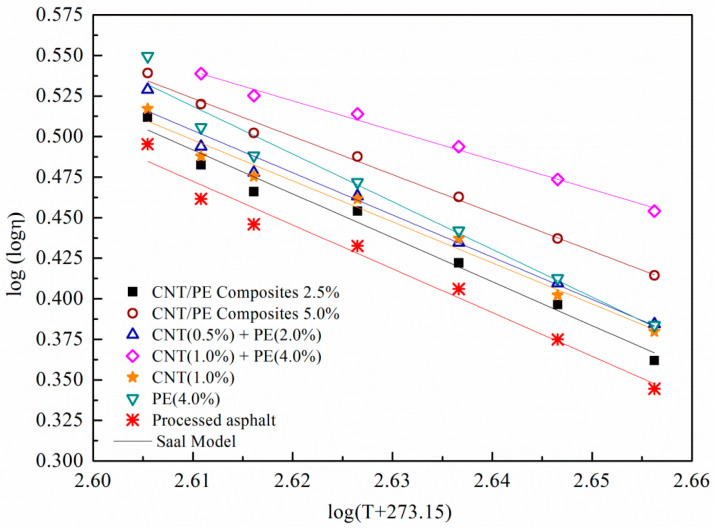
Temperature dependence of viscosity for various binders.

In the served temperature range of binder, the ideal binder is of high viscosity or sufficient deformation resistance to prevent rutting distress. Meanwhile, the binder is expected with sufficient low viscosity and good elasticity to relax the low temperature stress and to prevent thermo-cracking. Moreover, the binder should be of low viscosity and good workability in the high temperature range of pumping, handling, and paving. Thus, the relationship of viscosity with temperature of binder is a key technical parameter to evaluate the viscosity–temperature characteristics. Figure 2 illustrates the variation of viscosity with temperature for asphalt modified by CNT, PE, and the composites. Adding CNT or PE into the base asphalt increased the viscosity of the binder. CNT served as a filler for enhancing the friction of asphalt molecules in asphalt, whereas PE absorbs the light friction of asphalt giving rising to the hardening effect of the binder (physical distillation) [18,27]. The combined addition of CNT and PE significantly increase the viscosity of the modified asphalt in comparison to single modifier, especially for binder with CNT (1%) plus PE (4%). Whereas the melt mixing CNT/PE composites lead to a lower viscosity comparing to the combined method in the case of same dosage. Some researchers believed that orientational effects of CNT in asphalt contribute to the increased viscosity of the modified asphalt [28,29]. Due to the orientational effects of CNT, binders with combined addition of CNT and PE show higher viscosity comparing to samples with melt mixing CNT/PE composites. For the CNT/PE composites in the study, CNT were previously dispersed in PE matrix by melting blending method. The orientational effects of CNT weaken due to the coating of PE and this resulted in the decreased internal friction of CNT molecules. Consequently, binders with melt mixing CNT/PE composites show decreased viscosity.

As shown in Figure 2, log-log of viscosity has linear relationship with the logarithmic temperature for studied samples. The linear relationship can be described by Saal model, which is shown in Equation (1):

log log(*η*) = n − m log (*T* + 273.13) (1)

where *η* is Brookfield viscosity, mPa·s; *T* is temperature, °C; m and n are linear regression coefficients. The value of m indicates temperature susceptibility of viscosity. Higher absolute value of m means the more sensitivity of viscosity to temperature. The m values of Saal model fitting for various specimens were listed in Table 3. Adding CNT or PE decreased the sensitivity of viscosity to temperature for binder. The combined addition of CNT and PE leads to the least temperature susceptibility of viscosity. In addition, viscosity of binder with melt mixing CNT/PE composite is more sensitive to temperature changes, which is beneficial for the workability.

### 3.3. Dynamic Mechanical Properties Based on Oscillation Shear Test

#### 3.3.1. Black Diagrams

**Figure 3:**
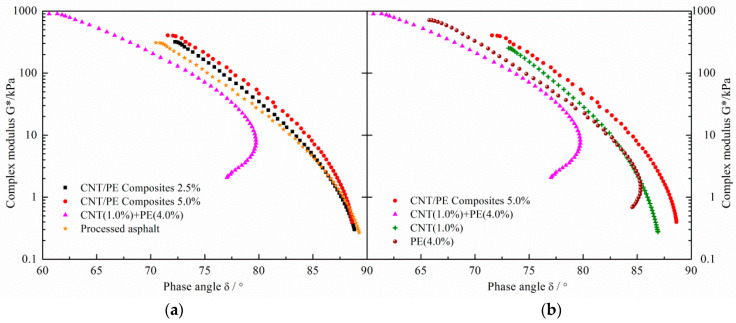
Black diagrams of asphalts with different CNT/PE modifier in temperature range from 30 to 90 °C, (**a**) comparison of processed asphalt and different CNT/PE modified asphalt, (**b**) comparison of PE, CNT and CNT/PE modified asphalt.

The effects of CNT, combined use of CNT and PE, and melt mixing CNT/PE composite on the mechanical properties of resulting binders were evaluated by complex modulus and phase angle based on dynamic oscillation shear test. Complex modulus and phase angle with a temperature range from 30 to 90 °C are plotted in black diagrams, which is a single graph displaying complex modulus as a function of phase angle regardless of frequency and temperature. Black diagrams are generally employed to identify discrepancies of experimental results and to determine thermo-rheological simplicity of modified asphalt. Black diagrams of asphalts with different CNT/PE modifiers in a temperature range from 30–90 °C are summarized in Figure 3.

As presented in Figure 3, CNT, combined use of CNT & PE and melt mixing CNT/PE composite caused the different viscoelastic properties of binders. Left graph in Figure 3 displays the difference in black diagrams of binders with CNT/PE composite, binder with CNT & PE and processed asphalt. A monotonic decrease of complex modulus with phase angle is observed for binders with CNT/PE composite and processed asphalt. The G–δ curves corresponding to CNT/PE composite and processed asphalt almost overlap at high temperatures (region of low G* & high δ), indicating a similar rheological behavior. Meanwhile, complex modulus increases and phase angle decreases as the increase of the CNT/PE composite at intermediate temperature. Notably, a hump appears in the curve of binder with 1.0% CNT & 4.0% PE, indicating a complex modulus threshold level (approximately 10 kPa) that separates two distinctive domains. For the domain below the threshold, phase angle decreases with complex modulus, corresponding to the increased stiffness and enhanced elasticity of binder at high temperatures. Thus, binder with 1.0% CNT & 4.0% PE shows an improvement of the rutting resistance potential comparing to sample with 5% CNT/PE composite. Such result indicates that the reinforcement of CNT is more obvious in the case of combination with PE polymer. Right graph in Figure 3 compares the difference in black diagrams of binders with CNT/PE composite, binder with CNT & PE, CNT modified asphalt and PE modified asphalt. A similar hump appears in the curve of typical PE modified asphalt while the monotonic trend was observed for 1% CNT modified asphalt. These comparisons imply that only adding CNT to improve the high temperature of binder is limited. It is noteworthy that high shear mixing was employed to prepare CNT modified asphalt. High shear mixing is not enough to obtain adequate dispersion of CNT in the binder matrix. The contribution of nanotubes to asphalt probably will be impaired due to the inadequate dispersion of CNT. However, high shear mixing is sufficient to obtain an adequate dispersion of CNT/PE masterbatch composites in the asphalt matrix. Therefore, the combined use of CNT and PE shows a significant enhancement of high temperature performance of binder, which may be attributed to synergistic reinforcement of both two constituents. Moreover, the monotonic decrease of curves for binders with CNT/PE composites indicate the inferior rutting resistance potential.

#### 3.3.2. Rutting Resistance Factor and Failure Temperature

**Figure 4:**
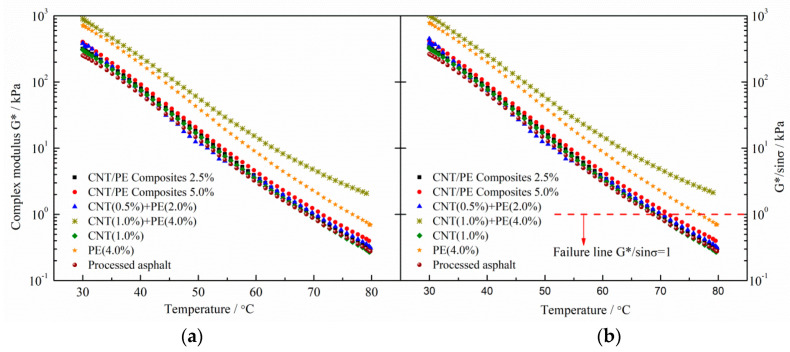
Rheological properties of asphalts with different CNT/PE modifier in the temperature range from 30 to 90 °C, (**a**) complex modulus G*, (**b**) anti-rutting factors G*/sin δ.

Owing to the visco-elastic properties of asphalt materials, rutting resistance of asphalt pavement is crucial at a high-service temperature. If the viscous behavior of asphalt is dominant, the binder is prone to creep under traffic loads. Complex modulus and G*/sinδ are key parameters to identify the resistance to permanent deformation of asphalt binder at high-service temperature. Thus, temperature sweep results within a wide temperature range for various samples are also shown in Figure 4. As displayed, only adding CNT resulted in a minor increase in complex modulus of binder, which maybe attributes to the inadequate dispersion of CNT in asphalt by means of high shear mixing. For binder with CNT/PE melt mixing composite, the increase in G* is limited in spite of its dosage from 2.5% to 5.0%. In terms of combined addition method, small dosage of CNT (0.5%) and PE (2%) caused a minor increase in G*. However, a significantly increasement is observed for binder with 1% CNT & 4% PE. In other words, the reinforcement of CNT is much more obvious when it is utilized together with common PE, even though they were mixed by the high shear method. Consequently, both CNT and PE lead to the enhancement of rutting resistance and such improvement is more evident by combined addition method. In addition, the CNT/PE melt mixing composite doesn’t display the synergistic reinforcement of both constituents. The failure temperatures can be calculated from G*/sinδ-T curves at the point of G*/sinδ = 1.0 kPa, which are listed in Table 4. The failure temperature verified the above viewpoint.

### 3.4. Creep and Recovery Behavior

The above high temperature performances based on dynamic shear in linear viscoelasticity range reveal the effect of different CNT/PE addition. Notwithstanding, the report [30] argued that rutting resistance G*/sinδ is inadequate to identify the creep behavior at high temperatures for modified asphalt, since permanent deformation is caused by the accumulated non-recoverable strain during creep. To overcome such limitations, the MSCR was proposed to identify the recoverable and non-recoverable strain response of binder when it is imposed by multiple shear loading and unloading cycles. Based on this consideration, MSCR test was conducted in the study to investigate the CNT/PE addition on permanent deformation behavior of the modified asphalt.

**Figure 5:**
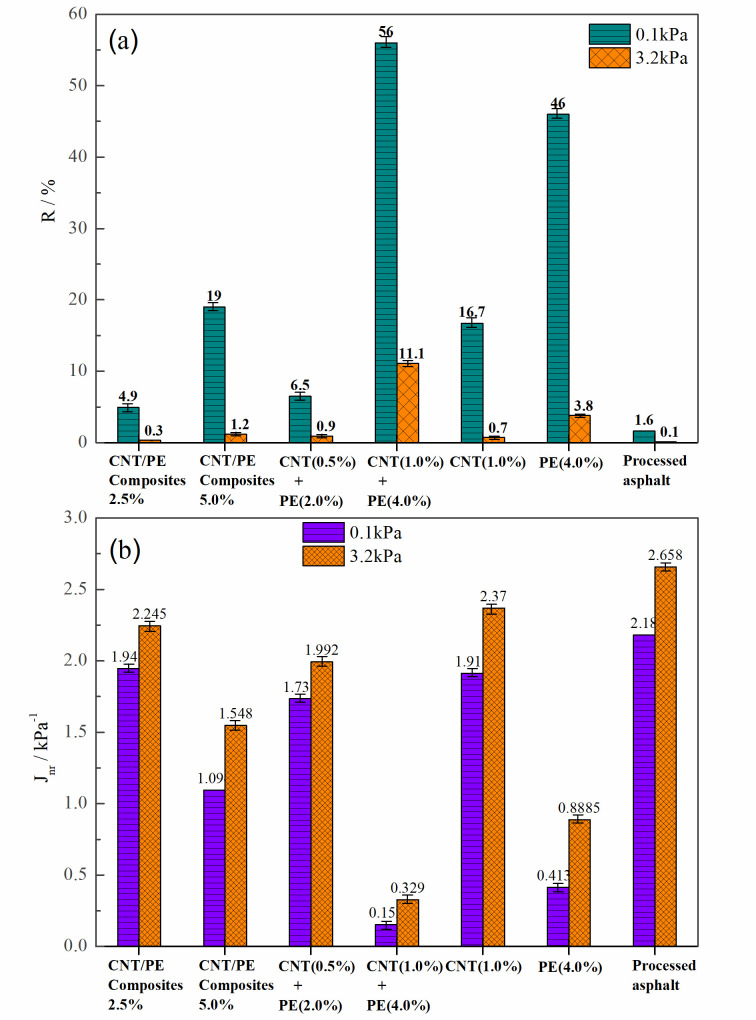
Average non-recoverable creep compliance and average percent recovery for asphalts with different CNT/PE, (**a**) average percent recovery R, (**b**) average non-recoverable creep compliance Jnr.

Figure 5 presents the MSCR results for asphalts with different CNT/PE: average percent recovery (R, Figure 5a) and average non-recoverable creep compliance (J_nr_, Figure 5b). Percent recovery shows the binder’s recovery ability, increased R corresponds to improved elastic response of asphalt. Meanwhile, Non-recoverable creep compliance, especially under the stress of 3.2 kPa, is well correlated with permanent deformation of pavement. Lower J_nr_ implies better resistance to permanent deformation. When the loading stress increases from 0.1 kPa to 3.2 kPa, a drastic decrease in R is observed for all studied sample and an increased J_nr_ is observed. When compared with processed asphalt, adding CNT leads to increased R and decreased J_nr_. Common PE modified asphalt (4%) also shows high R and low J_nr_, indicating good rutting resistance. For combined addition CNT & PE into asphalt, when the dosage increases from 0.5% CNT & 2.0% PE to 1.0% CNT & 4.0% PE, a significant increase in R and a great decrease in J_nr_ is found. The increase in R and decrease in J_nr_ are limited, when the dosage of CNT/PE composite increases from 2.5% to 5.0%. For the same dosage of CNT/PE, binders with melt mixing CNT/PE composite always present lower R and higher J_nr_ compared with the one with combined use of CNT & PE. Thus, the combined addition of CNT &PE for asphalt modification outperforms the CNT/PE melt mixing composites, which may be related to the above-mentioned synergistic reinforcement. A sonication procedure will be used in next research plan and the combined addition of CNT &PE for asphalt modification is expected to be more effective.

To identify the rutting resistance of binders, the AASHTO M332 specification [31] designates four different equivalent single axle load (ESAL) based traffic levels, including Standard (≤10 million ESALs), Heavy (>10 and ≤30 million ESALs), Very Heavy (≥30 million ESALs), and Extreme (≥30 million ESALs plus standing traffic loading). Upper limits on the J_nr3.2_ value were defined, i.e., 4.5 kPa^−1^ for the ‘S’ level, 2 kPa^−1^ for the ‘H’ level, 1 kPa^−1^ for the ‘V’ level, and 0.5 kPa^−1^ for the ‘E’ level. Hence, binder with 2.5% CNT/PE composite and binder with 1% CNT accord with ‘S’ level. Binder with 5% CNT/PE composite, binder with 0.5% CNT & 2.0% PE satisfy the ‘H’ level. PE modified asphalt (4%) satisfies the ‘V’ level. Only binder with 1.0% CNT & 4.0% PE satisfies the ‘E’ level, which shows great resistance to permanent deformation. The results indicate that the reinforcement of CNT is obvious when used in combination with adequate PE.

**Figure 6:**
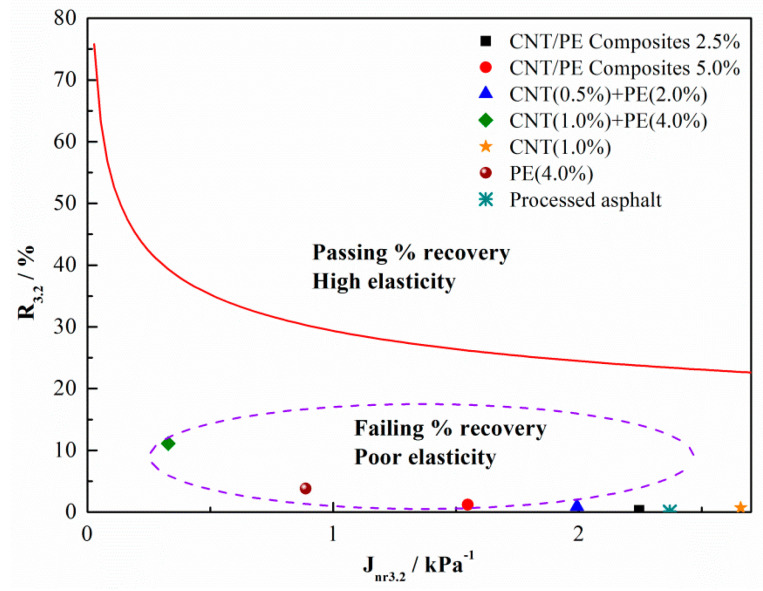
Analyses of MSCR test results: the plots of percent recovery R vs. Jnr at 3.2 kPa and the standard line.

Modification effect of various CNT/PE additives can be detected by evaluating the percent recovery, R_3.2_, along with the J_nr3.2_. The plots of percent recovery R_3.2_ vs. J_nr3.2_ and the standard line are presented in Figure 6. The relationship for detecting polymer modification was recommended in AASHTO TP70 [32], which is also called a standard line and can be described by Equation (2).

R = 29.371 (J_nr3.2_)^−0.2633^ (2)

The proposed standard curve marks the range of elasticity and is generally used to detect modification effect of modifiers, i.e., whether the binder passes or fails the percent recovery. Data points above the standard curve indicate binder has the sufficient delayed elastic response and has been modified with elastomeric polymers. Otherwise, data points below the standard curve indicate binders of poor elasticity. As presented in Figure 6, data points of all studied samples falls below the standard curve. This shows that the addition of CNT/PE leads to poor elasticity of binder and the modifiers did not endow asphalt with enough elasticity whether CNT or PE.

### 3.5. Low Temperature Fracture Behavior

Considering the positive effect of CNT/PE addition on the high temperature performance of asphalts, it is necessary to evaluate the low temperature performance of the studied binder. Since both CNT and PE increase the modulus and stiffness of binders, the studied binder has the risk of cracking initiation. Fracture behavior at low temperatures is crucial for binder, which predicts the cracking resistance and leads to the cracking propagation. In accordance with this consideration, the direct tension test (DTT) was carried out in the section to provide an understanding of the fracture resistance for asphalts with different CNT/PE at −12 °C.

**Figure 7:**
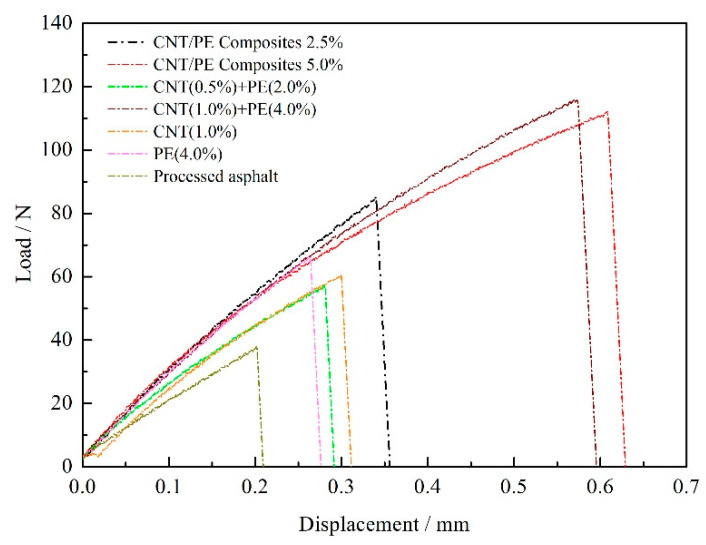
The relationship of load-displacement for asphalts with different CNT/PE at −12 °C.

**Figure 8:**
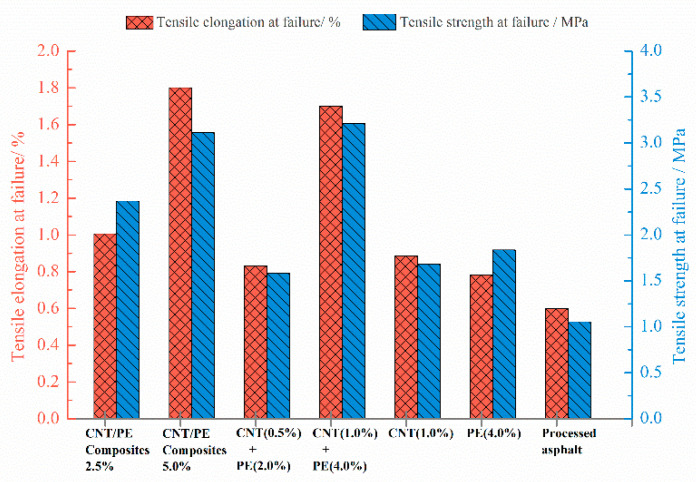
Tensile elongation and tensile strength at failure for various asphalts with CNT/PE at −12 °C.

The relationship of load-displacement was recorded during DTT for the tested samples, which is displayed in Figure 7. An approximate linear relationship between load and displacement is observed at the beginning, until reaching the maximum value, load drastically decreases to zero when fractured. However, the fracture point is very different for various samples. Overall, the addition of CNT, PE, or both leads to the increased breaking displacement. The breaking displacement of binder increases with the increase of CNT/PE addition. For the same content, binder with CNT/PE composite displays the higher fracture displacement comparing to the combined addition of CNT & PE. In order to quantitatively distinguish the fracture behavior of various samples, tensile elongation and tensile strength at break were calculated and the results are shown in Figure 8. As illustrated in Figure 8, processed asphalt has the lowest breaking tensile elongation and breaking strength. Compared with processed asphalt, adding CNT increases the tensile elongation of original binder (the improved cracking resistance), even though the CNT asphalt sample was mixed by high shear method. Literatures [20,32] reported that CNT do not affect the anti-cracking of aged binder and the pull-out behavior of CNT reinforced the interface. Thus, the reinforcement effect of CNT on phase interface may contribute to the improved breaking elongation of original binder in DTT. Common PE modified asphalt (4%) also shows a slight increase in breaking tensile elongation and breaking strength. The combined addition of CNT and PE causes enhanced breaking elongation and strength of binders, especially at the larger dosage in this study. Binder with the CNT/PE melt mixing composites presents the highest breaking tensile elongation at the same dosage. Both two types of CNT/PE modifiers benefit for the cracking elongation of original binder, meanwhile, the CNT/PE melt mixing blends outperform the combined addition of the two constituents. The reason could be illustrated as follows: the addition of CNT may reinforce the interface of PE phase and asphalt phase, which hinder the fracture potential of binder at low temperatures. The reinforcement effect of the nanotube in CNT/PE melt mixing blend is more obvious. Since the CNT is mixed previously with PE matrix, the linking effect and pull-out behavior of CNT/PE composite are more beneficial to the fracture resistance when it is dispersed in asphalt.

### 3.6. Aggregate-Binder Bonding Behavior

**Figure 9:**
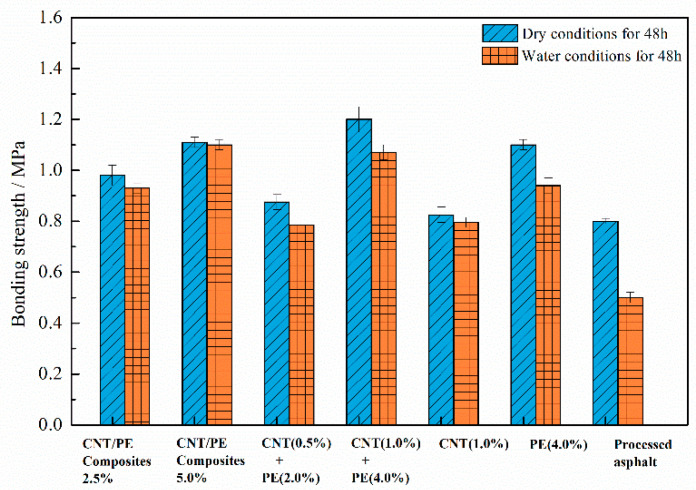
Binder bonding strength results in dry and water conditions for various CNT/PE asphalts.

To examine the effect of CNT on adhesion behavior of the modified asphalt, binder bonding strength between aggregate substrate and the studied binder was measured using Positest AT-A equipment in this section. Binder bonding strength results of studied samples in two conditions, i.e., a dry condition and a water condition, were presented in Figure 9. During the test, the limestone substrate was used to prepare the testing specimen of aggregate-binder. The sandwiched asphalt film between substrate and stub, meanwhile, the loaded pulling rate was 0.7 MPa/s. The temperature of dry conditioning was 20 °C with a humidity controlled below 30%, while in water conditions, the specimens were immersed in water bath at 20 °C for 48 h before testing.

Figure 9 illustrates that in dry conditions, enhanced bonding strength is observed for binders with CNT/PE additive as well as PE binder comparing to processed asphalt. Also, a minor increase in bonding strength can be seen for CNT modified asphalt comparing to processed asphalt, which may be related to the inadequate dispersion of CNT in asphalt by high shear method. Asphalt with combined addition of CNT (1.0%) & PE (4.0%) displayed the highest bonding strength, followed by binder with 5.0% CNT/PE composite as well as PE binder. The bonding results in dry conditions are mainly related to the stiffness of binder itself, where bonding strength is well in accord with in complex modulus and viscosity. For test after water conditioning, a reduction in bonding strength is observed. However, the decrease extent is different for various samples. The bonding strength of processed asphalt reduces more than 30% after water conditioning, while only a minor decrease in bonding strength is observed for CNT modified asphalt. This implies that CNT leads to improved moisture damage resistance of modified asphalt. Binders with CNT/PE composite show a small decrease in bonding strength when subjected to water conditioning, indicating good resistance to moisture damage. Binder with the combined addition of CNT and PE shows a larger decrease after moisture conditioning in bonding strength, comparing to binders with same dosage of CNT/PE composite. In other words, the CNT/PE composites endow asphalt with better resistance to moisture damage, in comparison with the combined addition method. The improved bonding strength and enhanced moisture damage resistance of binder with CNT may be interpreted as two aspects. Firstly, CNT shows strong adsorption ability to organic substance, especially asphalt components, because of its extreme large specific surface area. Thus, the CNT existing on the interface between asphalt and substrate reinforced the cohesion and bonding behavior. In addition, CNT displays high hydrophobicity and the dispersed CNT may hinder water penetration into bulk asphalt materials, which benefits the enhanced moisture damage resistance.

### 3.7. Micro-Morphology

The CNT/PE modified asphalt in this study is a typical composite material. The macroscopic performances of composite materials are influenced by any change in their micro-morphology, which in turn, can be altered by the change in morphology. To understand the above rheological properties, it is crucial to reveal micro- morphology of the studied samples.

**Figure 10:**
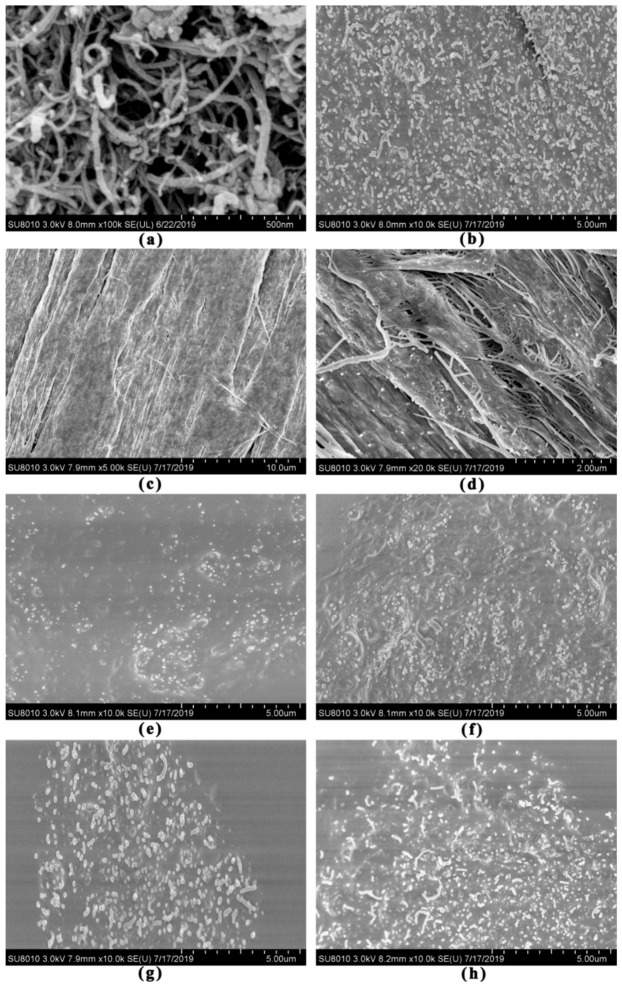
SEM images of various samples: (**a**) CNT; (**b**) Asphalt with 1% CNT; (**c**) CNT/PE melt mixing composites in 10μm; (**d**) CNT/PE melt mixing composites in 2μm; (**e**) Asphalt with 2.5% CNT/PE composites; (**f**) Asphalt with 5.0% CNT/PE composites; (**g**) Asphalt with 0.5% CNT + 2.0% PE; (**h**) Asphalt with 1.0% CNT + 4.0% PE.

Morphology was analyzed based on the SEM results. SEM images of CNT, CNT/PE composites and their modified asphalts are shown in Figure 10. As it has been presented in Figure 10a, CNT has an obvious tendency of aggregation that contact with each other to form the networks of agglomerations. This agglomeration was caused by the van der Waals force and thus, it is crucial to disperse the CNT aggregates in asphalt as uniformly as possibly in order to maximize the advantage of CNT modifier. Figure 10b shows the morphology of CNT modified asphalt prepared by high shear mixing. The CNT with rod like bright color can be identified. It can be seen that properly dispersed CNT in asphalt can be obtained with high shear mixing, but the dispersed CNT still remains dense and tends to stick together. The results indicate that the energy of high shear mixing is not enough to separate the single molecule from their aggregations, due to a high special surface area of CNT. Sonication procedure is suggested to be used in next research plan and the dispersion of CNT is expected to be more uniform. Figure 10c,d displays the SEM morphology of CNT/PE melt mixing composites in 10 μm and 2 μm. The dark flat area is PE and the bright long rod like area is the tube coated by polymer. The texture of CNT/PE composites appears to form ordered structure due to the very high aspect ratio of the CNT. The distribution of CNT in PE polymer matrix is basically uniform. Figure 10e,f shows the morphology of binder with 2.5%, 5.0% CNT/PE melt mixing composites respectively. The light dot is CNT and the rough area is PE phase. It could be seen that CNT and PE formed an evenly dispersion system on the whole for binder with 2.5% CNT/PE composites. This indicates that high speed shear mixing is enough to result in fairly well dispersion of CNT/PE composites in asphalt. Once the PE matrix is shredded into small pieces by mixer and distributed evenly in asphalt, the CNT is subsequent dispersed in asphalt. With the increase of CNT/PE composites in asphalt (binder with 5.0% CNT/PE composites), a rougher interface is observed and the dispersion of CNT also tends to be denser, which can be attributed to the improved high temperature performance. However, for the binder prepared with combined addition of CNT and PE, the dense and aggregated bright area of CNT could be seen in Figure 10g,h, which indicates the unevenly dispersion of CNT. Whereas the PE phase appears to be smoother in the method of combined addition of CNT and PE, which verifies the good dispersion of PE by high shear mixing. Last but not least, parts of CNT were distributed on the interface between PE phase and asphalt for the two types of CNT/PE modified asphalt, which reinforce the adhesion of interface. The researcher also reported the pull-out behavior of CNT or nano-fibers in polymer modified asphalt [33,34,35] and they believed the reinforcement effect of CNT attribute to pull-out behaviors. Therefore, the reinforcement of CNT to the interface accounts for the improved bonding behavior of the resulting binder.

### 3.8. Thermal Stability

**Figure 11:**
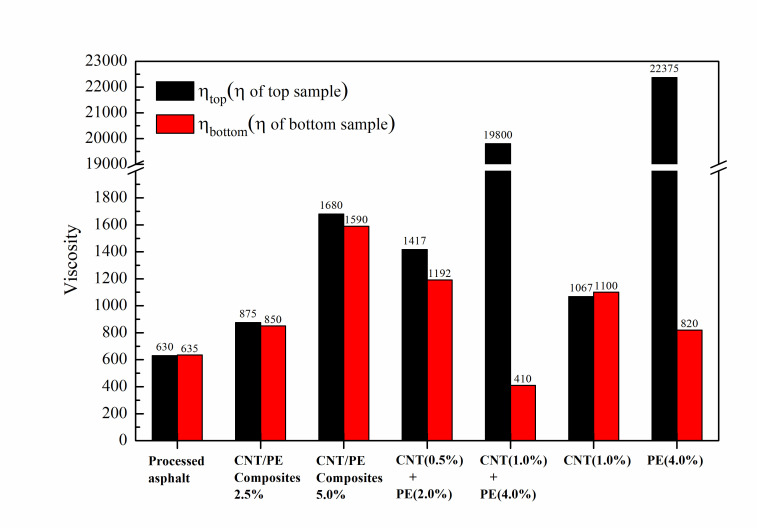

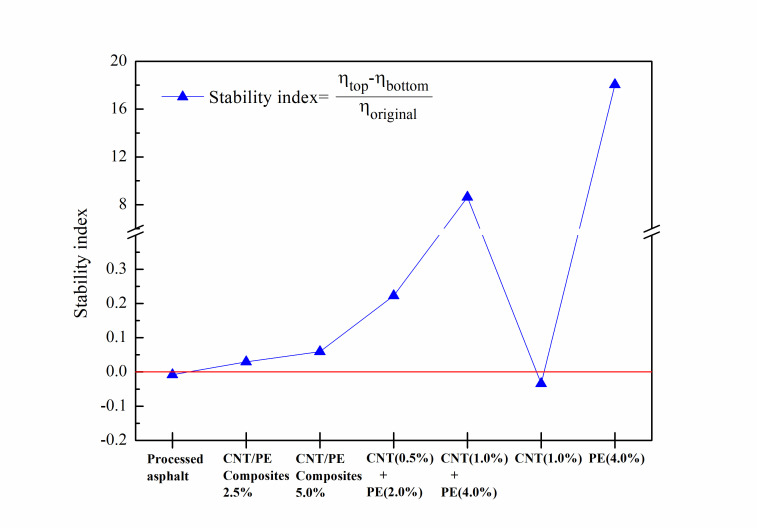
Viscosity of sample from top and bottom parts of the tube (**a**); and stability index for various samples (**b**).

The influence of CNT on thermal stability of the modified asphalt was investigated by conducting high temperature storage tests. Viscosity for samples obtained from the top and bottom sections of the tube after storage was measured using rotational viscometer at 135 °C. Moreover, the stability index, calculated from the difference in viscosity between top and bottom samples, was employed to quantitatively evaluate the effect of CNT on storage stability in the study. The results were presented in Figure 11a,b. As shown in Figure 11a, processed asphalt has almost the same value in viscosity for top and bottom sections. However, the difference in viscosity between top and bottom sample is quite small for asphalt with 2.5% CNT/PE composites. Such difference in viscosity becomes larger for asphalt modified with combined addition of 0.5% CNT & 2.0%PE. Meanwhile, a significant discrepancy in viscosity between top and bottom sample is observed for binder with 1.0% CNT & 4.0%PE. In the same dosage, by contrast, the difference in viscosity is much lower for asphalt with 5.0% CNT/PE composites. These results indicated that asphalt modified by CNT/PE composites shows quite good storage stability. The improved storage stability may be attributed to the reinforcement effect of CNT on the interface between polymer phase and asphalt matrix. Wang et al. [20] highlighted the contribution of pull-out behavior of CNT and its reinforcement effect on the interface. It is reasonable to deduce that the pull-out behavior of CNT/PE composite contributes to the improved storage stability of the modified asphalt, in which such the pull-out behavior can be verified in Figure 10. PE modified asphalt showed the most obvious difference in viscosity for top and bottom. Such a difference reduces for asphalt modified with CNT and PE. Thus, the incorporation of CNT can improve the storage stability of PE modified asphalt. Furthermore, the parameter, i.e., stability index, was defined as the ratio of viscosity difference after storing with original viscosity of fresh sample, in order to quantitatively characterize the stability. The value of stability index is closer to zero, the better storage stability is. The parameter of stability index for the studied samples is shown in Figure 11b. It can be seen that the value of stability index for asphalt with CNT/PE composite is lower than that of asphalt with CNT and PE, especially for the case of higher modifier content. The stability index of PE modified asphalt is very high while adding CNT can reduce the value of stability index. These results verify the improved storage stability for asphalt with CNT/PE composite and the contribution of CNT to PE modified asphalt. The improved storage stability is expected for CNT/PE composite modified asphalt when using the sonication preparation procedure [21].

## 4. Conclusions

This study provides a comprehensive understanding of the performance of a binder with CNT/PE composites. Two forms of CNT/PE modifiers, i.e., CNT/PE melt mixing composites and the combined addition of CNT & PE, were used for asphalt modification. The effect of CNT on rheological properties, adhesion behavior, morphology and storage stability of PE modified asphalt were investigated. The experimental results obtained in this paper indicate a positive effect of CNT/PE composites on performance of binder. A synergistic reinforcement of CNT and PE was found in this paper, which significantly contributes to the performance improvement of binders. The conclusions drawn from experimental results in this work are summarized in the following:

- The incorporation of CNT/PE leads to increased softening point, decreased penetration, and increased ductility of binder. Combined addition of CNT and PE into asphalt shows better physical properties than the melt mixing CNT/PE composite. In addition, the combined addition of CNT and PE significantly increase the viscosity of the modified asphalt in comparison to a single modifier, whereas the melt mixing CNT/PE composites lead to a lower viscosity compared to the combined method, indicating the improvement of workability.
- The combined use of CNT and PE shows a significant enhancement of high temperature performance of binder, which can be deduced by higher complex modulus, increased percent recovery, and reduced creep compliance. Whereas, the contribution of single CNT to high temperature performance of binder is limited (related to the dispersion of CNT in asphalt by high shear mixing), the reinforcement effect of CNT is more significant when combined with adequate PE. In terms of low temperature cracking resistance, the two types of studied CNT/PE modifiers benefit the cracking elongation of binder at low temperatures. Meanwhile, the CNT/PE composites outperform the combined addition of CNT and PE.
- The bonding behavior of the binder was improved by the CNT/PE modifiers. CNT/PE improves moisture damage resistance of modified asphalt. The CNT/PE melt mixing composites endow asphalt with better resistance to moisture damage in comparison with the combined addition method.
- From the prospective of micro-morphology, the dispersed CNT still remains dense and tends to stick together in CNT modified asphalt. Asphalt with CNT/PE composites formed an evenly dispersion system on the whole, since CNT is dispersed in PE melting matrix previously, high speed shear mixing is enough to result in fairly well dispersion of CNT/PE composites in asphalt. Notably, some CNT bridges on the interface between PE phase and asphalt for the two types of CNT/PE modified asphalt, which reinforces the adhesion of interface.
- The CNT/PE composites lead to the significantly improved storage stability of binder, while the storage stability of asphalt with combined CNT and PE is poorer than that of the binder with CNT/PE composites. The CNT/PE composites are recommended for asphalt modification due to its balanced performance (with better storage stability and lower rheological performance). Compared with PE modified asphalt, the incorporation of CNT in PE modified asphalt benefits storage stability. Last but not least, even though these results are positive, more investigations and further assessment of pavement, including performance of asphalt mixtures and road paving applications, need to be carried out. In any case, the conclusions in this work can be referred to by researchers in future.

## Figures and Tables

**Table 1:** Properties of used multi-wall CNT in the study.

Items	Measured Values
Average diameter/nm	10–20
Tube length/μm	5–15
Purity/%	≥92.0
Ash content/%	≤8.0
Specific surface area (BET)/m^2^∙g^−1^	220–260
Tap density/ g∙cm^−3^	0.01–0.06
Metal content/ppm	≤10

**Table 2:** Properties of HDPE.

Items	Measured Values
Density/g·cm^−3^	0.954
Crystallinity/%	86
Melt point/°C	130
Melt flow index (MFI)/g·(10 min)^−1^	0.8
Break elongation/%	>1000
Break strength/kg·cm^2^	390

**Table 3:** Parameters of Saal model fitting for various specimens.

Specimens	Absolute Value of m	n
CNT/PE Composites 2.5%	2.700	7.558
CNT/PE Composites 5.0%	2.355	6.670
CNT (0.5%) + PE (2.0%)	2.604	7.299
CNT (1.0%) + PE (4.0%)	1.821	5.292
CNT (1.0%)	2.529	7.099
PE (4.0%)	2.690	8.193
Processed asphalt	2.711	7.516

**Table 4:** Failure temperature for original binders with CNT/PE modifier.

Specimens	Fatigue Temperature/°C
CNT/PE Composites 2.5%	69.90
CNT/PE Composites 5.0%	71.50
CNT (0.5%) + PE (2.0%)	70.10
CNT (1.0%) + PE (4.0%)	87.50
CNT (1.0%)	68.95
PE (4.0%)	76.74
Processed asphalt	68.68
